# Upregulation of Trx alleviated high-glucose-induced Müller cell pyroptosis through ASK-1/Cav-1-mediated endoplasmic reticulum stress and autophagy

**DOI:** 10.3389/fimmu.2026.1747872

**Published:** 2026-02-27

**Authors:** Kaimin Bao, Xuebin Yu, Limin Wei, Yang Yu, Shiwen Zhong, Zhiyi Ren, Hongyang Chen, Li Kong, Xiang Ren, Hui Kong

**Affiliations:** 1 Department of Otorhinolaryngology of the Second Hospital, Dalian Medical University, Dalian, Liaoning, China; 2 Department of Histology and Embryology, College of Basic Medicine, Dalian Medical University, Dalian, Liaoning, China; 3 Key Laboratory of Reproductive Biology, Dalian Medical University, Dalian, Liaoning, China

**Keywords:** autophagy, Caveolin-1, diabetic retinopathy, endoplasmic reticulum stress, pyroptosis, thioredoxin

## Abstract

**Objectives:**

Diabetic retinopathy (DR) is a vision-threatening complication of diabetes. A high-glucose state induces endoplasmic reticulum stress (ERS) and autophagy in retinal Müller cells and further triggers pyroptosis, which ultimately promotes the progression of DR. Apoptosis signal-regulating kinase 1 (ASK1) and caveolin-1 (Cav-1) have been found to be closely associated with ERS and autophagy. Thioredoxin (Trx), a small-molecule protein, is essential for regulating cellular function. However, the regulatory mechanisms linking these molecules are not fully understood in DR. In this study, we investigated the role and mechanism of Trx in alleviating high-glucose-induced pyroptosis in Müller cells.

**Study design:**

Serum samples from patients with diabetes, diabetic mice, and Müller cells were used in the study.

**Results:**

*In vivo* and *in vitro*, high glucose can lead to increased expression of retinal inflammatory factors, morphological damage, and induction of cell pyroptosis. After high-glucose treatment, the expression of ERS- and pyroptosis-related genes increased. This process was reversed after Trx overexpression. Furthermore, autophagy was activated, and the number of TUNEL-positive cells decreased. These effects were reversed by Cav-1 inhibitor treatment.

**Conclusions:**

Trx overexpression could delay high-glucose-induced Müller cell pyroptosis by regulating ERS and autophagy via ASK-1/Cav-1, providing a new therapeutic target for DR treatment.

## Introduction

Diabetes mellitus (DM) is a metabolic disorder associated with hyperglycemia. According to recently published data from the International Diabetes Federation, the number of people with DM is expected to reach 783 million globally by 2045 (1). Prolonged hyperglycemia can damage various tissues and organs, including the eye, kidney, nerves, heart, and blood vessels, ultimately endangering patients’ health and life.

Diabetic retinopathy (DR) is a major ocular complication of DM and a leading cause of blindness and visual impairment, seriously affecting patients’ quality of life. Previous studies on the pathophysiology of DR have predominantly focused on vascular mechanisms, with most clinical manifestations involving retinal microvascular injury, such as microaneurysms, hemorrhages, and hard exudates (2). Increasing evidence indicates that retinal neurological dysfunction and vascular disruption may occur simultaneously, and in some cases, neuropathy—including damage to neurons and glial cells—may even precede vascular disruption (3). Therefore, exploring the mechanisms underlying retinal glial cell disorders may represent an effective strategy for treating DR.

The pathophysiological changes in DR involve a variety of causative factors, including blood-retinal barrier (BRB) disruption, disturbed glucose metabolism, oxidative stress (OS), endoplasmic reticulum stress (ERS), immunogenetics, and inflammation. Especially, inflammatory responses induced by high-glucose conditions are critical to the development and progression of DR (4–6). The inflammatory response triggered by metabolic changes in diabetes leads to damage of the retinal neurovascular unit, resulting in progressive retinal neuropathy (7, 8).

Müller cells are the major glial cells of the retina and the only cells that span its entire width, maintaining close contact with retinal blood vessels and neurons. Müller cells play a key role in regulating the inflammatory response in the retina (9). Therefore, finding the molecular mechanism or method to restore Müller cell homeostasis could be an effective strategy for DR treatment.

All intrinsic or extrinsic factors that disturb endoplasmic reticulum (ER) homeostasis lead to ERS, which interferes with the normal process of protein synthesis and modification and can result in the accumulation of misfolded or unfolded proteins. In studies of idiopathic pulmonary fibrosis, caveolin-1 (Cav-1) structural scaffold peptide (CSP) was found to alleviate ERS by inhibiting inositol-requiring enzyme 1α (IRE1α) (10). Recent studies have shown that ERS and the induced inflammatory responses contribute to the pathological basis and dysfunction of DM (11, 12). ERS stimulates cells to generate reactive oxygen species (ROS), which dissociates thioredoxin-interacting protein (TXNIP) from thioredoxin (Trx) in the nucleus; TXNIP then translocates from the nucleus into the cytoplasm to bind with NOD-like receptor protein 3 (NLRP3), ultimately activating the NLRP3 inflammasome (13). During this process, most retinal cells undergo pyroptosis, a mode of programmed cell death. This process, previously termed cellular inflammatory necrosis, depends on the formation of the inflammatory vesicle NLRP3 and proteins related to the gasdermin family. Pyroptosis is characterized by rupture of cell membranes, chromatin condensation, nuclear fragmentation, and the extensive release of cellular contents, including interleukin-1β (IL-1β) and interleukin-18 (IL-18) (14). In addition, interventions in IL-1 signaling and gene therapy targeting pyroptosis have received considerable attention for effectively controlling the inflammatory response in the eye (15).

Cav-1 is a major structural protein of the flask-shaped membrane domain (16) and regulates many cellular functions, including membrane trafficking, endocytosis, cell proliferation, and autophagy (17), an intracellular self-degradation process that is neuroprotective through the formation of double-membrane autophagosomes and their degradation via fusion with lysosomes, which are involved in removing proteins and damaged organelles (18). Cav-1 and autophagy have been found to participate in the development of DR, and Cav-1 promotes the formation of autophagosomes and autolysosomes by regulating insulin signaling (19).

Trx are small-molecule proteins that are widely present in the cytoplasm (Trx1) and mitochondria (Trx2) of living cells and regulate numerous cellular functions, including gene expression, antioxidant response, apoptosis, and cell proliferation. It is one of the most prominent antioxidants in mammals and is essential for signal transduction and cellular stress responses (20). In previous studies, Trx-1 exerted neuroprotective effects by inhibiting NLRP1-mediated neuronal pyroptosis in Alzheimer’s disease (AD) (21), and Trx modifies O-GlcNAc to retard apoptosis in retinal 661w cells (22).

Prior studies found that Trx in Müller cells in DR is primarily involved in oxidative stress (OS), ERS, and apoptosis (23, 24). However, it remains unclear whether Trx can regulate Müller cell pyroptosis, and the underlying mechanism has not been explored, indicating that Trx’s role in Müller cells is not fully understood. Therefore, Müller cells treated with HG (high glucose) will be used as a model to investigate the relationship and underlying mechanisms during DR, which may help to improve understanding and identify potential new therapeutic targets for DR in the clinic.

## Materials and methods

### Patients

A total of 108 patients with DM and 28 healthy individuals were admitted to the First Hospital of Dalian Medical University. Fasting venous blood was collected from diabetic patients and healthy people, centrifuged at 3.500 r/min for 10 min, and the serum/plasma samples were stored at – 80°C for subsequent assays. The criteria for inclusion were as follows: (1) meeting the diagnostic criteria for diabetic retinopathy of the Chinese Medical Association Diabetes Branch, (2) diagnosis confirmed by fundoscopy, and (3) no history of diabetes mellitus, hypertension, or related ocular diseases in the NC group. The exclusion criteria were as follows: (1) patients who had recently taken hormonal drugs or drugs that impair renal metabolism, (2) patients with a history of comorbid hypertension, (3) patients with a recent history of related ocular diseases, and (4) patients with a recent history of infection.

### Animal care

All experimental steps were performed according to the Institutional Guidelines for the Care and Use of Laboratory Animals, and the experimental protocols were approved by the Institutional Animal Care and Use Committee of the Experimental Animal Centre of Dalian Medical University. Male C57BL/6 mice, 6 weeks old, were obtained from the Experimental Animal Centre of Dalian Medical University and randomly divided into control and DM groups. The room temperature was 20 °C–25°C, the humidity was 55%–60%, and the mice were fed *ad libitum* with a 12-h day–night alternating environment for 1 week to allow adaptation. After this period, the control group was fed a basal diet, and the diabetic model group was fed a high-sugar, high-fat diet for 4 weeks, followed by a 12-h fasting period. The type 1 diabetic model group was injected intraperitoneally with streptozotocin (50 mg/kg) for 5 consecutive days (23), and the control group was injected with an equal volume of sodium citrate buffer. Fasting blood glucose was measured every other week after streptozotocin injection, and successful modeling was defined as a blood glucose level > 16.7 mmol/L along with insulin resistance (25, 26).

### Cell culture and reagents

The human Müller cell line was provided by the School of Basic Medical Sciences, Sun Yat-sen University. It was cultured in DMEM (Gibco, USA) medium containing 10% fetal bovine serum (FBS; FBS-S500, NEWZERUM, New Zealand), penicillin (100 units/mL), and streptomycin (100 µg/mL) at 37°C in 5% CO_2_. The medium was changed every 1 to 2 days. Cells were washed with Phosphate-Buffered Saline (PBS) before the experiment. The soybean glycosides (MCE, HY-N0019) were dissolved in DMSO at 1 mg/mL and stored at 4°C.

### Transfection

The plasmids pIRES2-EGFP-Trx and pIRES2-EGFP-LacZ were stably transfected into Müller cells according to the Lipofectamine 2000 instructions. Müller cell lines stably expressing Trx and Lacz were thus established. The stably transfected cells were identified by quantitative real-time (qPCR) and Western blot following expansion in culture. ASK1 small-interfering RNA (siRNA) from Genepharma, China (sense 5′-GCAUGGUACCUCAAGUCUATT-3′, anti-sense 5′-UAGACUUGAGGUACCAUGCTT-3′) was used to downregulate ASK1 expression in Müller cells.

### Cytokine assay

According to the manufacturer’s instructions, the IL-1β ELISA Kit (R&D, E20211101A) was used to detect the concentration of IL-1β in the serum of patients with diabetes. When the stop solution changed color from blue to yellow, the intensity was measured at 450 nm using a microplate reader. The concentration of IL-1β in the samples was calculated using a standard curve. The cytokines IL-1β, tumor necrosis factor-α (TNF-α), and interferon-γ (IFN-γ) from the serum of diabetic mice, patients with diabetes, and the medium of Müller cells were detected by flow cytometry using the kit (Micron Biological Cytokine Detection Kit, 3060030064).

### CCK-8 assay

The cells were seeded into 96-well plates (Guangzhou Jet Bio-Filtration Co. Ltd., China) at a density of 5,000 cells per well, and the peripheral wells were filled with sterile 0.1 M PBS. The cells were cultured at 37 °C in a 5% CO_2_ incubator and treated under different conditions. Subsequently, 10 μL of CCK8 reagent was added to each well, followed by incubation at 37 °C in 5% CO_2_ for 2 h. Absorbance was measured at 450 nm using a microplate reader.

### Western blot

Briefly, proteins were obtained using cell extraction kits supplemented with protease and phosphatase inhibitors (KeyGEN BioTECH, KGP903, China), stored at 4 °C for 15 min, and then centrifuged at 15,000 rpm for 20 min to collect the supernatants. Protein concentrations in each sample were measured using a BCA quantitative kit. Equal amounts of protein from different groups were prepared for SDS–polyacrylamide gel electrophoresis and transferred to polyvinylidene fluoride membranes. The membranes were blocked for 2 h in a nonfat milk diluted with Tris-Buffered Saline with Tween 20 (TBST) (0.5% Tween-20) at room temperature and then incubated overnight at 4 °C with the following primary antibodies: Trx (1:1,000, 14999-1-AP, Proteintech, China), glial fibrillary acidic protein (GFAP) (1:1,000, 60190-1-Ig, Proteintech), Txnip (1:1,000, 18243-1-AP, Proteintech), caveolin-1 (1:1,000, 16447-1-AP, Proteintech), NLRP3 (1:1,000, WL0263, Wanleibio), cysteinyl aspartate-specific proteinase (caspase-1) (1:1,000, Solarbio, China), apoptosis-associated speck-like protein containing a CARD (ASC) (1:1,000, 10500-1-AP, Proteintech, China) LC3 (1:1,000, 14600-1-AP, Proteintech), P62 (1:1,000, A7758, Abclonal), and Glyceraldehyde-3-Phosphate Dehydrogenase (GAPDH) (10494-1-AP, Proteintech). The membranes were washed three times with 1 × TBST for 15 min. Subsequently, they were incubated with goat antirabbit IgG (1:2,000, AS014, Abclonal) or goat antimouse IgG (AS003, 1:2,000, Abclonal) for 1 h at room temperature and washed three times with 1 × TBST for 15 min. The membranes were then exposed to X-ray film using an enhanced chemiluminescence system. The intensity of the bands was measured using LabWorks 4.5.

### Quantitative real-time polymerase chain reaction

According to the manufacturer’s instructions, total RNA was extracted from the cells after different treatments by using TRIzol (Takara, Japan), and cDNA was synthesized using the TransScript^®^ Uni All-in-One First-Strand cDNA Synthesis SuperMix for qPCR (One-Step gDNA Removal) Kit. Real-time PCR was performed using the PerfectStart^®^ Uni RT&qPCR Kit. The conditions for cDNA synthesis from RNA were as follows: 37 °C for 15 min, 85 °C for 5 s, and 4°C indefinitely. GAPDH was used as the internal reference for PCR amplification. The primer sequences were used as follows: Trx Forward: 5′-GTAGTTGACTTCTCAGCCACGTG-3′ and Reverse: 5′-CTGACAGTCATCCACATCTACTTC-3′; Cav-1 Forward: 5′-GACAGGGCGTTAAAAGCAGG-3′ and Reverse: 5′-TAGCAGTTCCAAGCACCAGT-3′); and GAPDH Forward: 5′-GTCTCCTCTGACTTCAACAGCG-3′ and Reverse: 5′-ACCACCCTGTTGCTGTAGCCAA-3′. The qRT-PCR amplification conditions were as follows: 94 °C for 30 s, 94 °C for 5 s, 55 °C for 15 s, 72 °C for 10 s for 40 cycles, 95 °C for 60 s, 55 °C for 30 s, and 95 °C for 30 s. The data were analyzed using the 2(–Delta Delta C(T)) method.

### Analysis of autophagic flux

The cells were infected with a tandem fluorescent monomeric Red Fluorescent Protein–Green Fluorescent Protein (mRFP-GFP)-tagged LC3 adenovirus (Beyotime, C3011, China). GFP and mCherry expression was observed using Keyence microscopy. Yellow spots (representing the merged GFP signal and RFP signals) indicate autophagosomes, while red spots (RFP signal alone) indicate autolysosomes. Autophagic flux was evaluated based on the GFP/mRFP color change.

### Transmission electron microscopy

The cells were treated with different conditions, collected, and fixed at 4 °C with glutaraldehyde, followed by fixation in 1% osmium tetroxide for 1 h. The samples underwent gradient ethanol dehydration, and the cells were embedded. Ultrathin sections were prepared and observed under a transmission electron microscope (JEM-2000EX*).

### TdT-mediated dUTP nick-end labeling

The cells were seeded and grown on glass coverslips and treated as indicated. After fixation with 4% paraformaldehyde for 30 min at room temperature, staining was performed, followed by three washes with PBS for 3 min each. Cells were permeabilized with 0.25% TritonX-100 for 5 min at room temperature and washed three times with PBS for 3 min each. Focal death was then detected using a one-step TdT-mediated dUTP nick-end labeling (TUNEL) kit (KGA7071) according to the manufacturer’s instructions. The number of positive cells was quantified under a microscope (Leica, Germany).

### Retinal morphological analysis

The eyeballs were fixed in Bouin’s fixative for 24 h, dehydrated through alcohol, and paraffin sections of the whole retina, including the optic disc, were prepared at a thickness of 5 μm and stained with hematoxylin–eosin (H&E). The total nuclear layer thickness of the retina was measured from the retinal pigment epithelium layer to the ganglion cell layer using Nikon software.

### Immunohistochemical fluorescence staining

Paraffin sections were melted in an oven at 60 °C, then dewaxed and hydrated using xylene and alcohol. Antigen retrieval was performed by adding 0.1% trypsin dropwise at 37 °C. Sections were blocked with goat serum at room temperature for 30 min, followed by incubation with the GFAP antibody (1:200, 60190-1-Ig, Proteintech) diluted in PBS at 37 °C for 2 h. The fluorescein isothiocyanate-labeled secondary antibody was diluted 1:1,000 and incubated at room temperature for 1 h. Finally, the sections were mounted with an antifluorescence quenching solution and observed under a fluorescence microscope to capture pictures.

### Bioinformatics analysis

In this study, data were obtained from the Gene Expression Omnibus database (GEO, https://www.ncbi.nlm.nih.gov/gds/). The single-cell RNA sequencing (scRNA-Seq) dataset GSE205123 was identified by searching study-related keywords such as “Müller cell”, “single-cell analysis”, “retina”, “diabetes”, etc. The data were divided into two groups: wild‐type mice (WT: BSK_WT; m/m) and the diabetic group (DB: BSK_DB; db/db). Kyoto Encyclopedia of Genes and Genomes (KEGG) and Gene Ontology (GO) enrichment analyses were performed on the selected target genes (data source: https://sangerbox.com). Gene screening criteria were p_val_adj < 0.05, pct.1 > 0.5. Protein–Protein Internet (PPI) networks were drawn using the STRING database (data source: https://cn.string-db.org/).

### Statistical analysis

The data are presented as the mean ± SD. The statistically significant differences between the two groups were analyzed using Student’s *t*-test. Comparisons among multiple groups were performed using one-way analysis of variance (ANOVA). All analyses were performed using GraphPad Prism software (Ver.8.0.2). A *p*-value < 0.05 was considered statistically significant (*α* = 0.05, Power = 0.8, Effect size = *d* = 0.5).

## Results

### Diabetes-induced glial activation of retinal Müller cells associated with retinal inflammation

**Figure 1:**
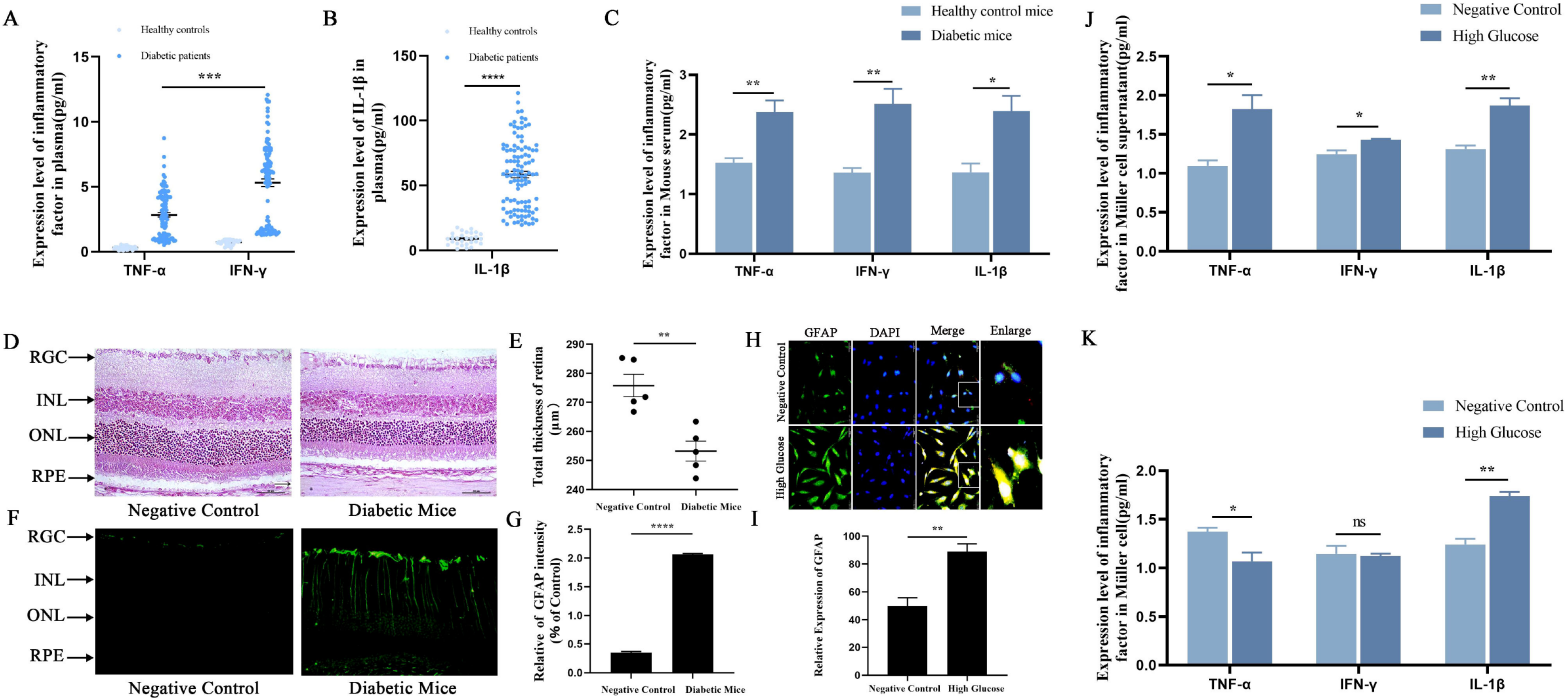
Diabetes-induced retinal Müller cells glial activation was related to retinal inflammation. **(A, B)** Flow cytometry was used to detect the levels of the inflammatory factors TNF-α and IFN-γ in the sera of diabetic patients, and ELISA was used to detect the levels of IL-1β. Healthy controls (*n* = 28) and diabetes (*n* = 108). **(C)** Flow cytometry was used to detect the levels of the inflammatory factors TNF-α and IFN-γ in serum samples from diabetic mice, and ELISA was used to detect the levels of IL-1β. Healthy control mice (*n* = 5) and diabetic mice (*n* = 5). In particular, IL-1β was closely related to pyroptosis. **(D)** Morphological changes in the retina of diabetic mice were observed by HE staining, where the scale bar is 50 µm. **(E)** The total retinal thickness was measured in diabetic mice. Healthy control mice (*n* = 5) and diabetic mice (*n* = 5). **(F, G)** The expression of GFAP was detected by immunofluorescence staining in diabetic mice. Healthy control mice (*n* = 3) and diabetic mice (*n* = 3). **(H, I)** The expression of GFAP was detected by immunofluorescence staining in Müller cells after high-glucose treatment. NC group (*n* = 3) and HG group (*n* = 3). **(J, K)** Flow cytometry was used to detect the levels of the inflammatory factors TNF-α, IFN-γ, and IL-1β in Müller cell culture supernatants and Müller cells after high-glucose treatment. NC group (*n* = 3) and HG group (*n* = 3). The data are expressed as the mean ± SD. Statistically significant differences between the two groups were analyzed using Student’s *t*-test. ^*^p < 0.05; ^**^p < 0.01; ^***^p < 0.001.

To investigate whether diabetes-induced inflammation produced by retinal Müller cells is related to glial cell activation, we examined inflammatory factors in human samples, mouse samples, and Müller cells, as well as the expression of GFAP in mouse and Müller cells, using ELISA and flow cytometry. As shown in **Figures 1A–C**, the levels of inflammatory factors TNF-α, IFN-γ, and IL-1β in the serum of diabetic patients and diabetic mice were significantly increased compared with the control group. In addition, the levels of TNF-α, IFN-γ, and IL-1β in Müller cells and their culture medium *in vitro* were also detected, with results shown in **Figures 1J, K**. Moreover, morphologic analysis of the retina using H&E staining revealed that total retinal thickness was reduced in diabetic mice compared with the control group (**Figures 1D, E**). Observation of GFAP expression in the retina using immunohistofluorescence staining indicated that GFAP expression was elevated *in vivo* and *in vitro*; its expression increased in the DM group compared with the control group (**Figures 1F–I**). These results suggest that Müller cells in a high-glucose environment lead to increased inflammatory factors and high GFAP expression, which may accelerate neurodegeneration by disrupting the vascular barrier, driving neovascularization, and ultimately causing irreversible vision damage.

### Bioinformatics analysis of gene regulation and underlying mechanisms involved in high-glucose-induced pyroptosis in Müller cells

To explore the relationship between Trx and ERS, autophagy, and focal death in Müller cells, bioinformatics was performed to examine their correlations. As shown in **Supplementary Figures S1A, B**, 11,781 cells were screened according to the Seurat criteria, of which 6,313 cells were from the WT group and 5,468 cells originated from the DB group. Based on retinal marker genes reported in the literature as screening criteria, cell annotation identified the following cell types with corresponding marker genes: cone cells (Opn1mw+), bipolar cells (Vsx2+, Pax6−), ganglion cells (Thy1+, Pax6+), Müller cell (Vsx2+, Pax6+, Rlbp1+), and Microglia Cell (Cx3cr1+), which were visualized using violin plots (**Supplementary Figure S1A**) and tSNE (**Supplementary Figure S1B**). Genes associated with ERS, cellular autophagy, and focal death-related enrichment pathways were selected, yielding 1,628 genes relevant to this study. The enriched 1,628 genes were screened using single-cell sequencing data, and five genes (DB group/WT group) related to this study were further identified: Txn1 (Trx-1), Hspa5 (GRP78), Cav-1, Map1lc3b (LC3II), and Txnip (**Supplementary Figures S1C–G**). The genes screened by single-cell sequencing were then combined with Trx, Txnip, ASK1, glucose-regulated protein 78 (GRP78), microtubule-associated protein1 light chain 3-II (LC3II), Sequestosome 1 (P62), NLRP3, etc., which are closely related to the subject study, for protein interaction network PPI mapping (data source https://cn.string-db.org/) (**Supplementary Figure S1J**). *In vitro*, Western blot for ERS-, autophagy-, and pyroptosis-related proteins showed that, compared with the control group, the high-glucose group exhibited significantly increased expression of the ERS proteins GRP78 (**Supplementary Figures S1K, L**), IRE1 (**Supplementary Figures S1K, M**), and C/EBP homologous protein (CHOP) (**Supplementary Figures S1K, N**); the autophagy proteins LC3II (**Supplementary Figures S1O, Q**) and P62 (**Supplementary Figures S1O, R**); and the pyroptosis-related proteins NLRP3 (**Supplementary Figures S1S, V**), caspase-1 (**Supplementary Figures S1S, W**), ASC (**Supplementary Figures S1S, X**), and Txnip (**Supplementary Figures S1S, U**). In contrast, the expression levels of Trx (**Supplementary Figures S1S, T**) and Cav-1 (**Supplementary Figures S1O, P**) were downregulated, which further validated the bioinformatics analysis.

### The effect of Trx on high-glucose-induced pyroptosis in Müller cells

**Figure 2:**
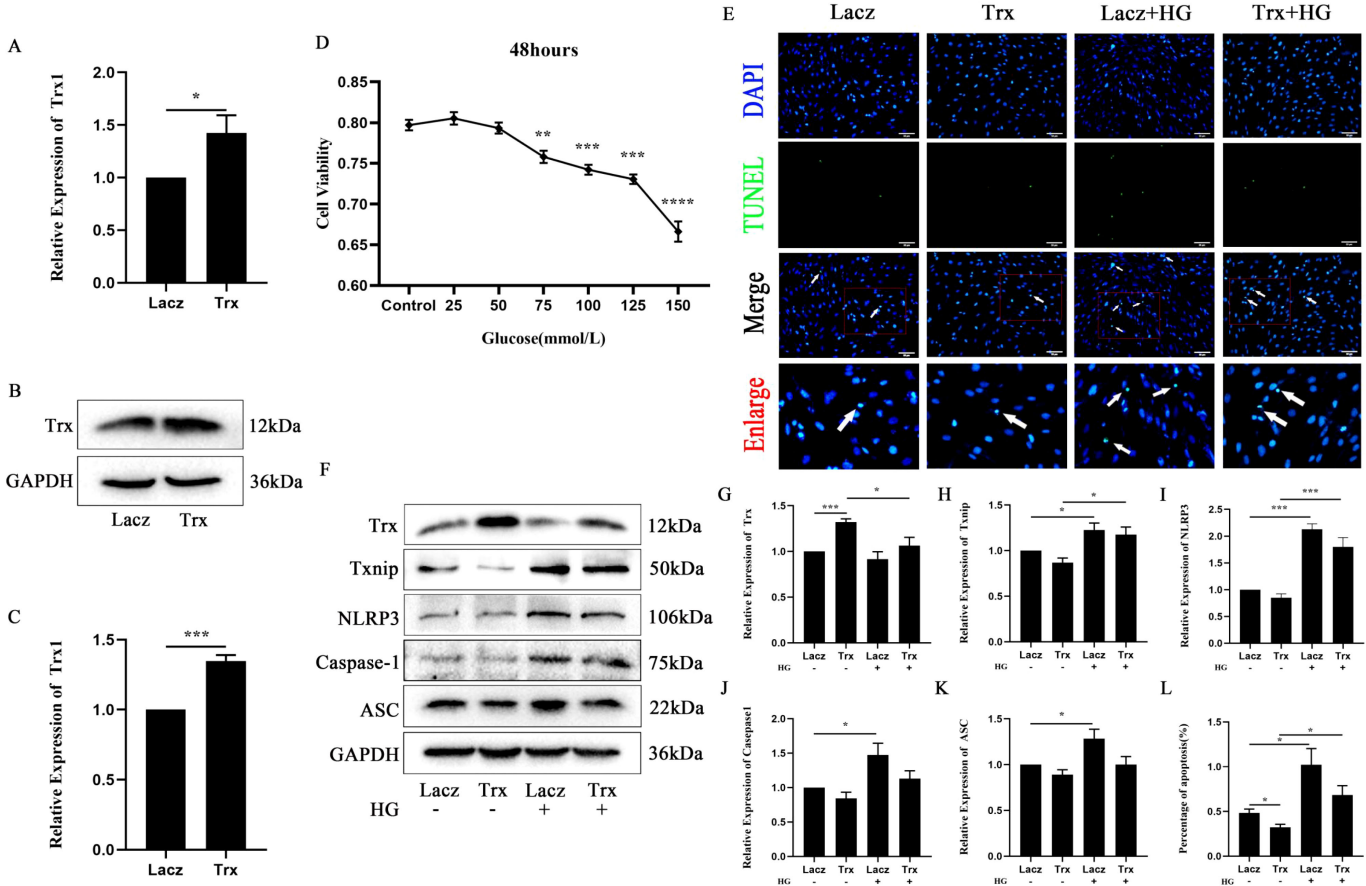
The effect of Trx on high-glucose-induced pyroptosis in Müller cells. qPCR (*n* = 8) and Western blot **(B**, **C)** were used to detect the expression of Trx in Müller cells after stable transfection (*n* = 5). **(D)** The optimal concentration and duration of high-glucose treatment in Müller–Lacz cells were determined using the CCK8 assay (*n* = 4). The effect of Trx on pyroptosis analysis in Müller cells treated with/without high glucose was analyzed by TUNEL staining **(E**, **L)** (*n* = 3). Western blot analysis was used to detect the expression of Trx **(F**, **G)** (*n* = 5), Txnip **(F**, **H)** (*n* = 3), NLRP3 **(F**, **I)** (*n* = 5), caspase-1 **(F**, **J)** (*n* = 5), and ASC **(F**, **K)** (*n* = 3) in Müller cells with Trx overexpression with/without high-glucose treatment. The data are expressed as the mean ± SD. Statistically significant differences between two groups were analyzed using Student’s *t*-test, and comparisons among multiple groups were performed using one-way analysis of variance (ANOVA). ^*^p < 0.05; ^***^p < 0.001.

To explore the effect of high glucose on Müller cell pyroptosis, cells were treated with high-glucose medium. To investigate the role of Trx in high-glucose-induced Müller cell pyroptosis, a Trx-overexpressing cell line and a parallel experimental group (Müller–Lacz cells) were used. As shown in **Figures 2A–C**, the expression of Trx was detected at both the gene and protein levels in Müller cells. The results of qPCR (**Figure 2A**) and Western blot analysis (**Figures 2B, C**) showed that Trx expression was increased after transfection compared with the control group. The optimal concentration and duration of high-glucose treatment in Müller–Lacz cells were determined using the CCK8 assay. The results showed that a significant decrease in Müller–Lacz cell viability occurred at a high-glucose concentration of 75 mM after 48 h (**Figure 2D**). The effect of high Trx expression on HG-induced Müller cell pyroptosis was analyzed, and the expression of Trx, Txnip, and pyroptosis-associated proteins was evaluated by Western blot. As shown in **Figures 2F–K**, the expression of Txnip (**Figure 2H**), NLRP3 (**Figure 2I**), caspase-1 (**Figure 2J**), and ASC (**Figure 2K**) was decreased, whereas the expression of Trx (**Figure 2G**) was increased in Müller–Trx cells compared with Müller–Lacz cells after high-glucose treatment. As shown in **Figures 2E, L**, TUNEL staining results demonstrated a significant decrease in the number of positively stained cells in Müller–Trx cells compared with Müller–Lacz cells after high-glucose treatment. These results suggest that changes in the expression of the redox system Trx/Txnip can be induced in response to high glucose, leading to the initiation of cellular pyroptosis, and that Trx overexpression can alleviate high-glucose-induced pyroptosis in Müller cells.

### The effect of Trx on high-glucose-induced ERS and autophagy in Müller cells

**Figure 3:**
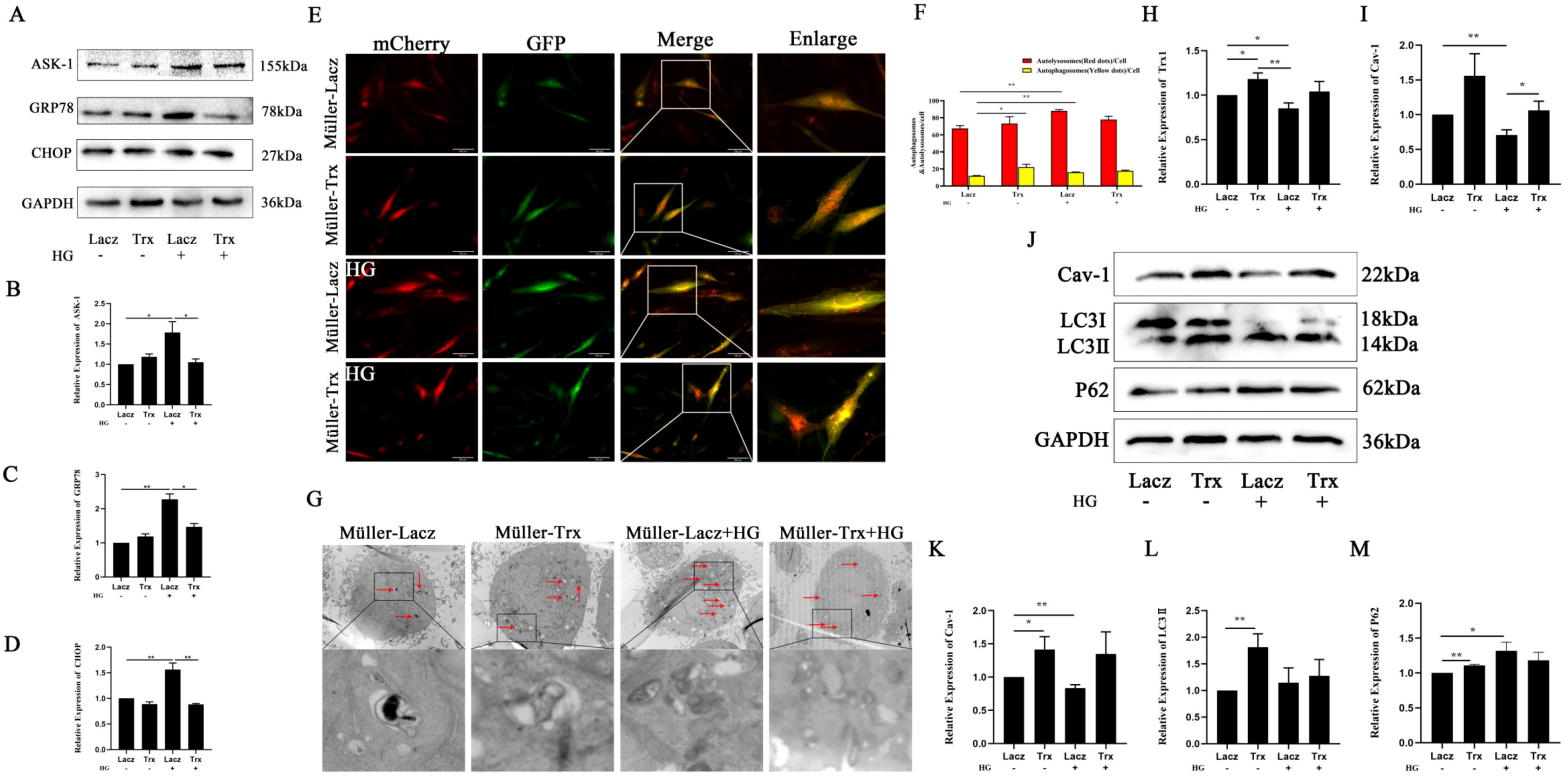
The effect of Trx on high-glucose-induced ERS and autophagy in Müller cells. **(A)** Western blot detection of Trx expression and ERS-related proteins ASK-1 **(A, B)** (*n* = 3), GRP78 **(A, C)** (*n* = 3), and CHOP **(A, D)** (*n* = 3). **(E, F)** The effect of Trx on autophagic flux in Müller cells under high-glucose conditions was monitored by mCherry-GFP-LC 3 dynamics (*n* = 3). **(G)** Transmission electron microscopy of autophagosomes in Müller cells under high-glucose conditions with Trx overexpression. **(H)** The effect of Trx overexpression on *Trx1* gene expression in Müller cells under high-glucose conditions (*n* = 8). **(I)** The effect of Trx overexpression on *Cav-1* gene expression in Müller cells under high-glucose treatment (*n* = 8). Western blot analysis was used to detect Trx expression of autophagy-related proteins Cav-1 **(J, K)** (*n* = 9), LC3 **(J–L)** (*n* = 8), and P62 **(J**, **M)** (*n* = 4). The data are expressed as the mean ± SD. Statistically significant comparisons among multiple groups were performed using one-way analysis of variance (ANOVA). ^*^p < 0.05; ^**^p < 0.01.

The effect of Trx on the presence of ERS and autophagy in high-glucose-induced Müller cells was investigated. In **Figures 3A–D**, ERS-related proteins and ASK1 were detected by Western blot. As shown in **Figures 3E, F**, to investigate the effect of Trx on high-glucose-induced autophagic flow in Müller cells, mCherry-GFP-LC3 was used to track the formation and degradation of autophagosomes. The results showed that the Müller–Lacz+HG group exhibited a significant increase in red and yellow spots compared with the Müller–Lacz group, and high Trx expression further increased the number of yellow spots in the cells. In addition, Müller cells treated with high glucose were collected, and the ultrastructure of intracellular autophagosomes was observed under a transmission electron microscope. The results showed that the number of autophagosomes in cells treated with high glucose was significantly higher than that in the group without high-glucose treatment, whereas the number of autophagosomes in Müller cells decreased after Trx overexpression (**Figure 3G**). Moreover, the gene expression of Trx-1 and Cav-1 was detected by qPCR (**Figures 3H, I**). As shown in **Figures 3J–M**, the expression of Cav-1 and the autophagy-related proteins LC3II and P62 was detected by Western blot. The results revealed that, in the Müller–Lacz+HG group compared with its control group, Cav-1 expression was significantly reduced, whereas LC3II and P62 expression was elevated. In contrast, in the Müller–Trx+HG group compared with the Müller–Lacz+HG group, the expression of Cav-1 and LC3II was elevated, while P62 expression was reduced. These results suggest that a high-glucose environment induces ERS and autophagy in Müller cells; however, Trx overexpression alleviates ERS and autophagy, possibly through ASK1- and Cav-1-mediated regulation of this process.

### Trx overexpression alleviates high-glucose-induced pyroptosis via ASK1-mediated ERS in Müller cells

**Figure 4:**
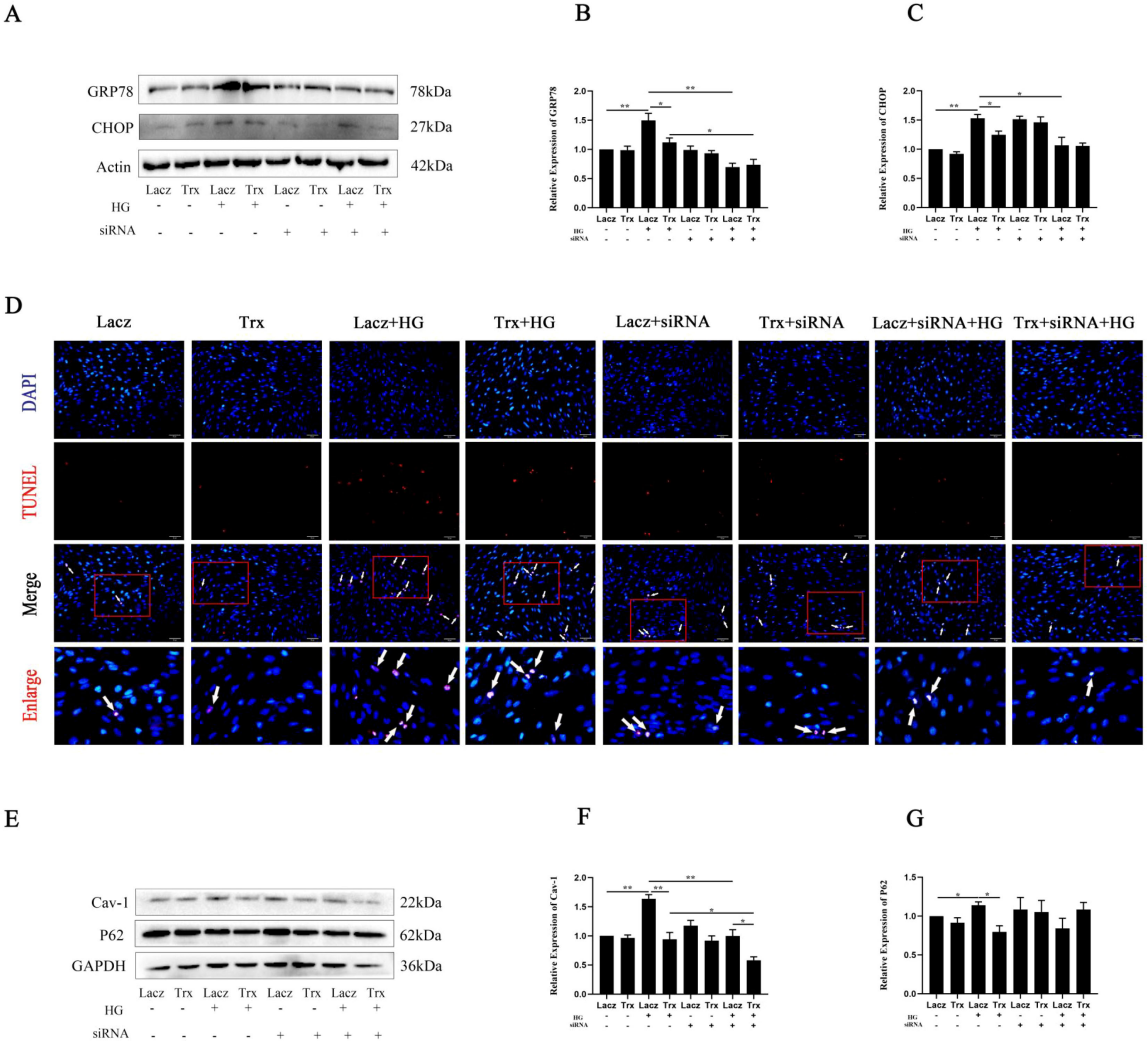
Trx overexpression alleviates high-glucose-induced pyroptosis via ASK1-mediated ERS in Müller cells. **(A–C)** The protein expression levels of CHOP and GRP78 were detected by Western blot after ASK1 siRNA treatment in Müller–Trx cells under high-glucose conditions (*n* = 3). **(D)** Pyroptosis analysis of Trx-overexpressing and ASK1 siRNA-treated Müller cells was performed by TUNEL staining (*n* = 3). **(E–G)** The protein expression levels of Cav-1 and P62 were detected by Western blot after ASK1 siRNA treatment in Müller–Trx cells under high-glucose conditions (*n* = 3). The data are expressed as the mean ± SD. Statistically significant comparisons among multiple groups were made using one-way analysis of variance (ANOVA). ^*^p < 0.05; ^**^p < 0.01.

To investigate whether overexpression of Trx regulates high-glucose-induced cellular pyroptosis through ASK-1-mediated ERS, the expression of GRP78 and CHOP was examined. As shown in **Figures 4A–C**, the expression of GRP78 and CHOP was significantly decreased in Müller–Trx cells after ASK1 siRNA transfection under high-glucose treatment. TUNEL staining was performed to assess the effect of ASK1 siRNA treatment on pyroptosis in Müller cells under high-glucose conditions. The results showed that the cellular pyroptosis rate of Müller–Trx–HG–siRNA was reduced compared with that in the Müller–Lacz–HG–siRNA group, suggesting that Trx overexpression alleviated high-glucose-induced Müller cell pyroptosis by regulating ASK-1 (**Figure 4D**). Moreover, the expression of Cav-1 and P62 was also examined, and the results are shown in **Figures 4E–G**.

### Effect of Cav-1 on Trx overexpression to regulate autophagy during high-glucose-induced Müller cell pyroptosis

**Figure 5:**
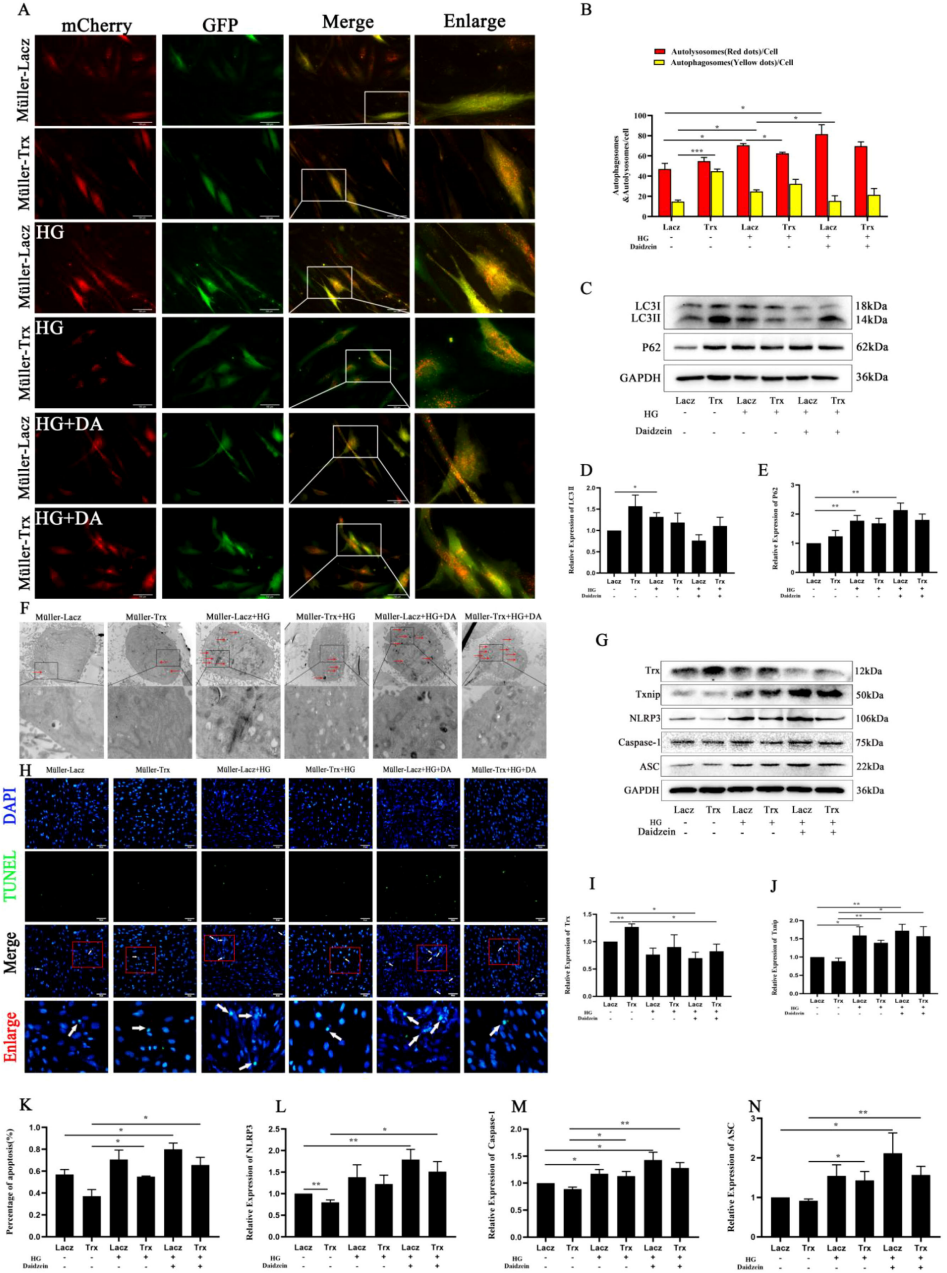
Effect of Cav-1 on Trx overexpression in regulating autophagy during high-glucose-induced Müller cell pyroptosis. **(A, B)** The effect of Cav-1 on Trx overexpression in delaying high-glucose-induced autophagy was detected by mRFP-GFP-LC3 in Müller cells with/without Daidzein treatment (*n* = 3). Western blot was used to detect the expression of LC3 **(C, D)** (*n* = 4), P62 **(C, E)** (*n* = 4), in Müller cells with/without Daidzein treatment. **(F)** Transmission electron microscopy was used to observe autophagosomes in Müller cells with/without Daidzein treatment. Pyroptosis analysis of Müller cells under high glucose with/without Daidzein treatment was detected by TUNEL staining **(H, K)** (*n* = 3). Western blot was used to detect the expression of Trx **(G, I)** (*n* = 4), Txnip **(G, J)** (*n* = 4), NLRP3 **(G**, **L)** (*n* = 6), caspase-1 **(G**, **M)** (*n* = 7), and ASC **(G**, **N)** (*n* = 9) in Müller cells treated with/without Daidzein under high-glucose conditions. The data are expressed as the mean ± SD. Statistically significant comparisons between multiple groups were determined using one-way analysis of variance (ANOVA). ^*^p < 0.05; ^**^p < 0.01; ^***^p < 0.001.

To investigate the association between Trx overexpression and the regulation of high-glucose-induced cellular pyroptosis and autophagy, autophagic flux was examined. As shown in **Figures 5A, B**, changes in autophagic flux were detected using mCherry-GFP-LC3. Cells were pretreated with daidzein (Cav-1 inhibitor). Compared with the Müller–Trx+HG+daidzein group, the Müller–Lacz+HG+daidzein group showed an increased number of yellow spots and a decreased number of red spots. Furthermore, transmission electron microscopy (TEM) was used to observe the formation of autophagic vesicles in Müller cells. As shown in **Figure 5F**, the number of autophagosomes in Müller–Lacz cells was significantly increased compared with the control group after high-glucose treatment; however, the number of autophagosomes in Müller–Trx cells was decreased compared with that in Müller–Lacz cells. However, this process was reversed after daidzein treatment. To further investigate whether Trx-regulated Cav-1 affects autophagy, the expression of Cav-1 in the Müller–Trx+HG group treated with daidzein treatment was compared with that in the Müller–Trx+HG group and was found to be reduced. In addition, the expression of LC3 (**Figures 5C, D**) and P62 (**Figures 5C, E**) in the Müller–Trx+HG group treated with daidzein treatment was increased compared with that in the Müller–Trx+HG group. These results suggest that a high-glucose environment induces autophagy in Müller cells, whereas inhibition of Cav-1 exacerbates DR by promoting autophagosome accumulation and impaired autophagic lysosome degradation. This pathological process can be reversed by high Trx expression.

As shown in **Figures 5H, K**, pyroptosis was detected by TUNEL staining, and the number of positive cells was increased in Müller–Lacz cells compared with the control group after high-glucose treatment. This increase was inhibited in Müller–Trx cells. However, the number of positive cells was also increased in Müller–Trx cells after high-glucose treatment with daidzein. Moreover, Western blot results showed that the expression of Txnip (**Figures 5G, J**), NLRP3 (**Figures 5G, L**), caspase-1 (**Figures 5G, M**), and ASC (**Figures 5G, N**) was increased in the Müller–Lacz+HG group compared with the Müller–Lacz group, and expression was reduced in the Müller–Trx+HG group compared with the Müller–Lacz+HG group. This process was reversed after daidzein treatment. Trx expression of Trx was also detected (**Figures 5G, I**). These results indicate that Trx overexpression alleviates high-glucose-induced pyroptosis via Cav-1-mediated autophagy in Müller cells.

## Discussion

DR is one of the most prominent neurovascular complications of diabetes and a leading cause of blindness. Uncontrolled hyperglycemia can lead to retinal damage and even blindness, making early detection and intervention critical for preventing DR. In this study, by collecting serum, retinal Müller cell supernatants, and cell suspensions from the retinas of clinical diabetic patients and mice, we detected a significant increase in the expression of the inflammatory factors TNF-α, IFN-γ, and IL-1β, suggesting that DR is closely associated with the development of inflammation. GFAP is considered a marker of retinal Müller cell damage (27), which can aid in early-stage DR diagnosis. We also found that a high-glucose environment led to glial cell activation and glioblast formation, accompanied by increased GFAP expression. In diabetic mice, the total retinal thickness was significantly reduced, and the fluorescence intensity of GFAP in the retina and Müller cells was markedly increased, indicating that DR is associated with gliosis of retinal Müller cells.

Chronic hyperglycemia is one of the main causative factors of DR and induces alterations in glucose metabolic pathways, such as the polyol pathway, in which glucose is reduced to sorbitol by aldose reductase. The impermeability of sorbitol causes it to accumulate in retinal cells, leading to cellular osmotic damage and subsequent oxidative stress (28). Previous studies have shown that hyperglycemia increases intracellular ROS, inducing OS and inflammation in retinal cells, along with activation of the TXNIP/NLRP3 pathway (29).

Trx is a multifunctional protein with a molecular weight of 12 kDa and is widely distributed in the human body. Trx dysfunction is associated with cancer, neurodegenerative diseases, and cardiovascular diseases (30, 31). Our previous study found that Trx inhibited ASK1 activation and effectively suppressed ERS under a high-glucose environment (21). Txnip can be activated and induce NLRP3 complex formation, promoting inflammatory responses (32). However, autophagy can inhibit NLRP3-mediated pyroptosis by degrading damaged organelles, leading to a reduction in pyroptosis (15, 33). Txnip inhibits both the expression and antioxidant effects of Trx by interacting with it. Previous studies have also shown that Trx plays a crucial role in alleviating diabetes-induced retinal cell death (22), suggesting that Trx could be a key target for diabetic retinopathy treatment through its anti-inflammatory and antioxidant effects.

scRNA-Seq technology is capable of analyzing gene expression at single-cell resolution, which is more accurate than other sequencing methods that average all data. It can also be used to specifically analyze a particular cell or even a subpopulation of cells. In recent years, the application of scRNA-Seq technology in diabetic retinopathy (DR) research has increased steadily, and studies have successfully isolated most types of retinal cells and identified marker genes for each cell by analyzing single-cell data from C57BL/6J mice of different ages (34). In this study, we analyzed and validated the results of the *in vitro* experiments based on a single-cell sequencing dataset of the mouse retina related to DR as reported in the literature, and we further examined the results of the exploratory study to determine gene expression. The findings were combined to validate the study results and analyzed to identify potential key targets of Trx in pathways related to alleviating diabetes-induced Müller cell damage. Key genes relevant to this study were analyzed using bioinformatics, and their interrelationships with ERS, autophagy, pyroptosis, Trx, and Cav-1 were examined via a PPI network. This analysis revealed that Cav-1 is closely linked to ERS and pyroptosis through autophagy, which was further validated *in vitro*. Under high-glucose conditions, the expression of ERS-associated proteins GRP78, IRE, and CHOP was significantly increased; Cav-1 expression was decreased; autophagy-related proteins LC3II and P62 expression were significantly upregulated; Trx expression was reduced; and the expression of pyroptosis-related proteins NLRP3, caspase-1, ASC, and Txnip was significantly elevated.

Based on the research background, the present study was conducted to investigate the mechanisms of pyroptosis, autophagy, and ERS in retinal Müller cells under high-glucose conditions using Müller cells with Trx overexpression. The expression of LC3II, P62, NLRP3, caspase-1, ASC, and ERS-related proteins was increased in Müller cells under high-glucose conditions, and Trx overexpression in these cells reversed this process. These findings suggest that Trx attenuates pyroptosis by regulating cellular autophagy and ERS in a high-glucose environment. However, the relevant mechanisms have not been fully explored.

To explore the mechanisms by which Trx and Cav-1 are correlated with focal death, autophagy, and ERS in retinal Müller cells under a high-glucose environment, further analyses were conducted. First, to examine the correlation between Trx-mediated alleviation of ERS and autophagy impairment and ASK1 and Cav-1 in Müller cells, ASK1 expression was inhibited using siRNA. The results showed that ASK1 exerted a negative regulatory effect and that Trx alleviated HG-induced ERS in Müller cells through ASK1. Although the autophagy-related protein P62 showed a corresponding regulatory trend, no significant differences were observed among the experimental groups, suggesting that ASK1 may not be a direct target of Trx in the regulation of autophagy. Secondly, to determine whether Trx regulates autophagy by mediating Cav-1 expression, experiments were performed using a Cav-1 inhibitor. The results indicated that inhibition of Cav-1 suppressed the expression of autophagy-related proteins. In summary, these findings suggest that Cav-1 acts as a downstream pathway through which Trx alleviates high-glucose-induced cellular pyroptosis by activating autophagy.

Studies have reported that the pathogenesis of Cav-1 in the eye and other related diseases is beginning to be elucidated. Cav-1 is expressed in a variety of retinal cells, including retinal endothelial cells, Müller cells, and retinal pigment epithelial cells (35), and it plays a key role in regulating retinal neuroinflammation and BRB permeability. Based on prelaboratory proteomics studies, diabetic mice were found to exhibit downregulated Cav-1 expression in retinal Müller cells compared with wild-type mice. In contrast, retinal Müller cells in mice with Trx overexpression showed increased Cav-1 expression, suggesting a possible positive regulatory relationship between Trx and Cav-1. On the other hand, Wu et al. demonstrated that Cav-1 promotes the formation of autophagic lysosomes and autophagic vesicles, indicating a potential role of Cav-1 in autophagy activation (36). Furthermore, autophagy removes damaged organelles and inhibits activation of the inflammatory vesicle NLRP3 (37). Therefore, in this study, we found that the expression of LC3 and P62 was increased, the expression of Cav-1 was decreased, and the expression of NLRP3, caspase-1, ASC, and ERS-related proteins was increased in a high-glucose environment *in vitro*. In addition, the results of TEM and the mCherry-GFP-LC3 autophagic flux assay demonstrated that Trx could activate autophagy to alleviate the onset of focal death. These results suggest that Trx plays a protective role in the retina by inhibiting autophagosome formation and accelerating lysosome degradation during DR, thereby alleviating cellular pyroptosis.

In our study, Cav-1 inhibition only partially reversed the effects of Trx, as the analysis focused on how Trx may maintain autophagic function through TXNIP/NLRP3 activation after Cav-1 inhibition. However, Trx is a multifunctional antioxidant protein, and its protective effects involve multiple signaling pathways, such as activation of the Nrf2 pathway or regulation of cell proliferation and apoptosis through the MAPK pathway. These pathways may remain functional after Cav-1 inhibition. In diabetic complications, the regulatory network of Trx is tissue-specific, and Cav-1 overexpression has a protective effect on the mitochondrial fission–mitochondrial autophagy axis, thereby alleviating diabetic cognitive dysfunction (38). In studies of diabetic nephropathy (DN), Trx binds to TXNIP, blocks NLRP3 activation, and reduces IL-1β release, thereby alleviating diabetic podocyte and glomerular injury (39). Given the tissue-specific regulatory network of Trx, future therapies should combine precision targeting (e.g., combined Cav-1 modulation in DR) and antioxidant strategies (e.g., TXNIP inhibition) to avoid the limitations of single-pathway interventions.

In summary, all findings are summarized in **Supplementary Figure S2**. In future studies, research could be conducted using Müller cells or mice with Cav-1 gene knockout, as well as Trx-overexpressing Müller cells or mice, to confirm the mechanism by which Trx-regulated, Cav-1-activated cellular autophagy mitigates ERS and pyroptosis in a high-glucose environment. Although the Cav-1 inhibitor Daidzein was used in our experiments, this drug has inherent limitations, including multiple targets of action. Regarding future research directions for Trx, transgenic mice with retinal tissue-specific or systemic Trx overexpression could be constructed in animal models. In addition, Trx overexpression could be achieved in adult animals (e.g., rats, nonhuman primates) using adeno-associated virus (AAV) or lentiviral vectors, thereby bringing the research closer to clinical intervention scenarios. On the other hand, regarding future directions of Trx research, gene-editing technologies could be utilized to achieve precise retinal-specific upregulation of Trx, thereby avoiding exogenous gene insertion. In addition, combination therapies that integrate Trx overexpression with anti-inflammatory drugs or mitochondria-targeted antioxidants may provide new therapeutic avenues for the clinical treatment of DR. In future studies, female animals will also be included to determine whether the current findings are generalizable across sexes and to explore potential sex-specific mechanisms. These findings suggest that Trx-based strategies may represent a beneficial approach for alleviating diabetic retinopathy and may provide new therapeutic targets and strategies for the clinical treatment of DR. Based on these findings, approaches such as lifestyle interventions or dietary intake of foods that induce Trx overexpression may be considered to alleviate retinal Müller cell pyroptosis in a high-glucose environment.

## Data Availability

The original contributions presented in the study are included in the article/**Supplementary Material**. Further inquiries can be directed to the corresponding authors.

## Supplementary material

Supplementary Figure 1 Bioinformatics analysis and *in vitro* validation of the regulation and mechanism of genes related to high glucose-induced Müller cell pyroptosis. **(A)** Stacked VlnPlot of cell biomarker. **(B)** All cell clusters identified by T-sne. The enriched genes were screened using single-cell sequencing data, and five genes (DB group/WT group) related to this study were further identified: Txn1 (Trx-1) **(C)**, Hspa5 (GRP78) **(D)**, Cav-1 **(E)**, Map1lc3b (LC3II) **(F)**, and Txnip **(G)**. **(H)** GO Circle plot. **(I)** GO Bubble plot. **(J)** PPI net. Western blot was used to detect the expression of Müller cell ERS-related proteins GRP78 **(K, L)**, IRE1 **(K, M)**, CHOP **(K, N)** in response to high glucose stimulation. Western blot was used to detect the expression of Cav-1 **(O, P)**, LC3-II **(O, Q)**, P62 **(O, R)**, Trx **(S, T)**, Txnip **(S, U)**, NLRP3 **(S, V)**, Caspase-1 **(S, W)**, ASC **(S, X)** in Müller cells after high glucose treatment. The data are expressed as the mean ± SD (n=3 for each group). The statistically significant differences between the two groups were analyzed using Student’s t-test. *P<0.05, **P<0.01, ***P<0.001.

Supplementary Figure 2 The summary of Up-regulation Trx alleviated high glucose-induced Müller cell pyroptosis.
