# Supplementary material for: Upregulation of Trx alleviated high-glucose-induced Müller cell pyroptosis through ASK-1/Cav-1-mediated endoplasmic reticulum stress and autophagy
Source: Front Immunol. 2026 Feb 27;17:1747872. doi: 10.3389/fimmu.2026.1747872 (PMC12983411; doi:10.3389/fimmu.2026.1747872)
Supplement: Supplementary Figure 1 — Bioinformatics analysis and in vitro validation of the regulation and mechanism of genes related to high glucose-induced Müller cell pyroptosis. (A) Stacked VlnPlot of cell biomarker. (B) All cell clusters identified by T-sne. The enriched genes were screened using single-cell sequencing data, and five genes (DB group/WT group) related to this study were further identified: Txn1 (Trx-1) (C), Hspa5 (GRP78) (D), Cav-1 (E), Map1lc3b (LC3II) (F), and Txnip (G). (H) GO Circle plot. (I) GO Bubble plot. (J) PPI net. Western blot was used to detect the expression of Müller cell ERS-related proteins GRP78 (K, L), IRE1 (K, M), CHOP (K, N) in response to high glucose stimulation. Western blot was used to detect the expression of Cav-1 (O, P), LC3-II (O, Q), P62 (O, R), Trx (S, T), Txnip (S, U), NLRP3 (S, V), Caspase-1 (S, W), ASC (S, X) in Müller cells after high glucose treatment. The data are expressed as the mean ± SD (n=3 for each group). The statistically significant differences between the two groups were analyzed using Student’s t-test. *P<0.05, **P<0.01, ***P<0.001. [file DataSheet1.docx]

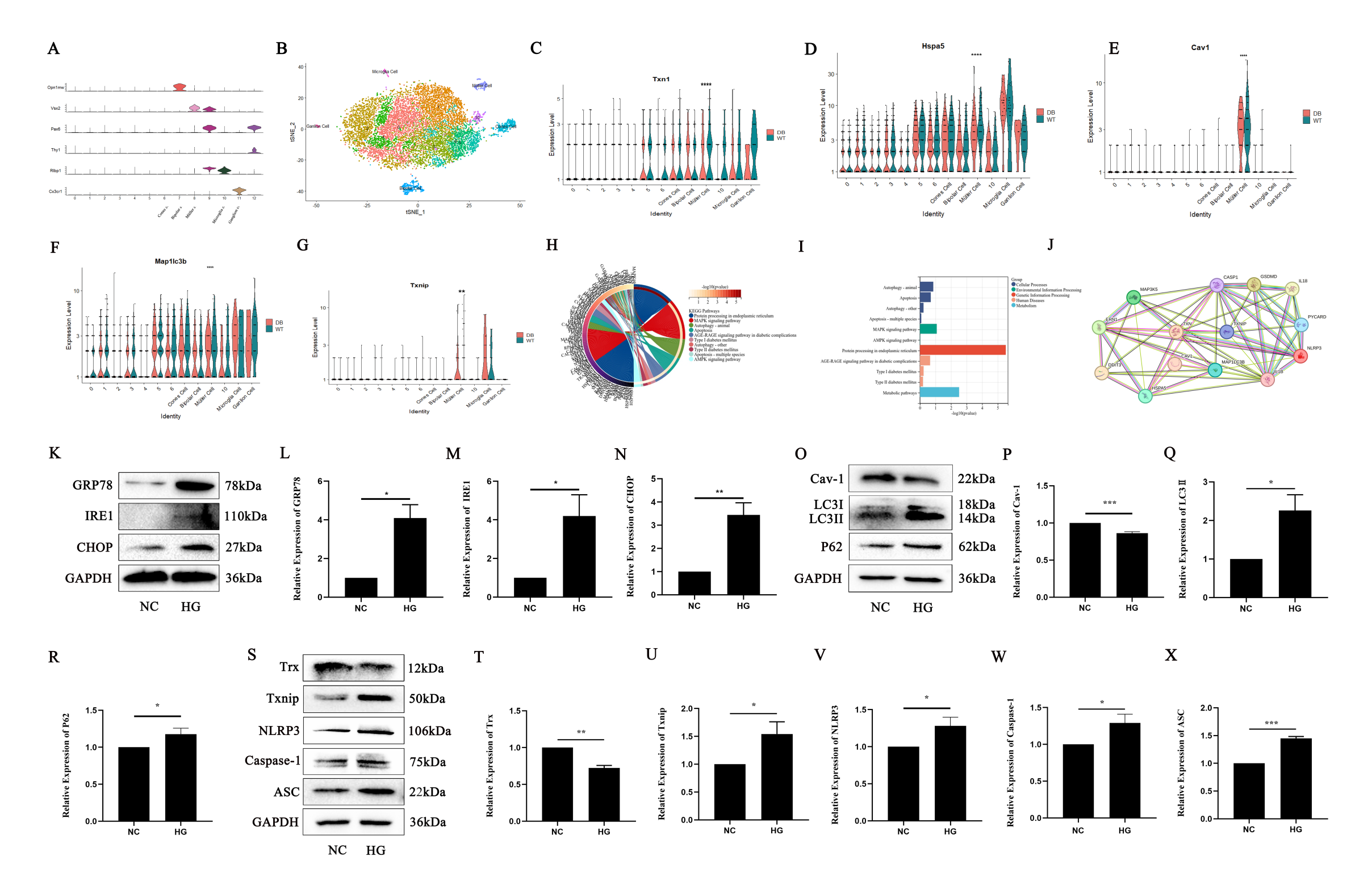


Supplemental figure 1

**Supplemental** **Figure 1** Bioinformatics analysis and in vitro validation of the regulation and mechanism of genes related to high glucose-induced

Müller cell pyroptosis. (A)Stacked VlnPlot of cell biomarker. (B) All cell clusters identified by T-sne. The enriched genes were screened using

single-cell sequencing data, and five genes (DB group/WT group) related to this study were further identified: Txn1 (Trx-1) (C), Hspa5 (GRP78) (D),

Cav-1 (E), Map1lc3b (LC3II) (F), and Txnip (G). (H) GO Circle plot. (I) GO Bubble plot. (J) PPI net. Western blot was used to detect the expression

of Müller cell ERS-related proteins GRP78 (K) & (L), IRE1 (K) & (M), CHOP (K) & (N) in response to high glucose stimulation. Western blot was used

to detect the expression of Cav-1 (O) & (P), LC3-II (O) & (Q), P62 (O) & (R), Trx (S) & (T), Txnip (S) & (U), NLRP3 (S) & (V), Caspase-1 (S) & (W),

ASC (S) & (X) in Müller cells after high glucose treatment. The data are expressed as the mean±SD (n=3 for each group). The statistically significant

differences between the two groups were analyzed using Student's t-test. *P<0.05, **P<0.01, ***P<0.001.


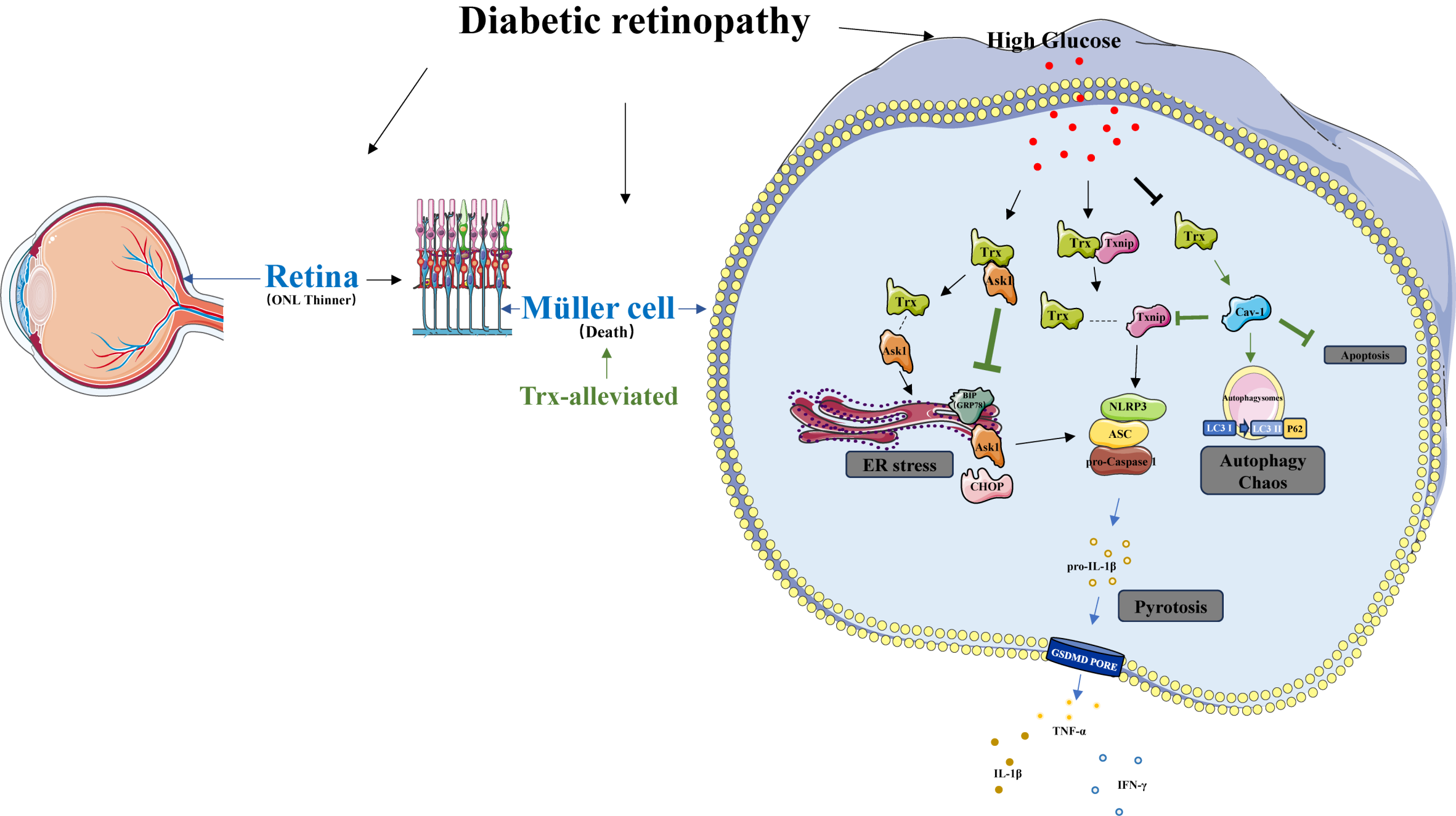
**Supplemental Figure 2** The summary of Up-regulation Trx alleviated high glucose-induced Müller cell pyroptosis.

Supplemental figure 2
